# The Influence of Ozonated Olive Oil-Loaded and Copper-Doped Nanohydroxyapatites on Planktonic Forms of Microorganisms

**DOI:** 10.3390/nano10101997

**Published:** 2020-10-10

**Authors:** Wojciech Zakrzewski, Maciej Dobrzynski, Joanna Nowicka, Magdalena Pajaczkowska, Maria Szymonowicz, Sara Targonska, Paulina Sobierajska, Katarzyna Wiglusz, Wojciech Dobrzynski, Adam Lubojanski, Sebastian Fedorowicz, Zbigniew Rybak, Rafal J. Wiglusz

**Affiliations:** 1 Department of Experimental Surgery and Biomaterial Research, Wroclaw Medical University, Bujwida 44, 50-345 Wroclaw, Poland; wojciech.zakrzewski@student.umed.wroc.pl (W.Z.); maria.szymonowicz@umed.wroc.pl (M.S.); adam.lubojanski@student.umed.wroc.pl (A.L.); zbigniew.rybak@umed.wroc.pl (Z.R.); 2 Department of Conservative Dentistry and Pedodontics, Wroclaw Medical University, Krakowska 26, 50-425 Wroclaw, Poland; maciej.dobrzynski@umed.wroc.pl; 3 Department of Microbiology, Wroclaw Medical University, Chalubinskiego 4, 50-368 Wroclaw, Poland; joanna.nowicka@umed.wroc.pl (J.N.); magdalena.pajaczkowska@umed.wroc.pl (M.P.); sebastian.fedorowicz@student.umed.wroc.pl (S.F.); 4 Institute of Low Temperature and Structure Research, Polish Academy of Sciences, Okolna 2, 50-422 Wroclaw, Poland; s.targonska@intibs.pl (S.T.); p.sobierajska@intibs.pl (P.S.); 5 Department of Analytical Chemistry, Wroclaw Medical University, Borowska 211 A, 50-566 Wroclaw, Poland; katarzyna.wiglusz@umed.wroc.pl; 6 Student Scientific Circle at the Department of Dental Materials, School of Medicine with the Division of Dentistry in Zabrze, Medical University of Silesia in Katowice, Akademicki Sq. 17, 41-902 Bytom, Poland; wojt.dobrzynski@wp.pl

**Keywords:** Cu^2+^ ions, ozonated olive oil, hydroxyapatite, antimicrobial activity, microorganisms

## Abstract

The research has been carried out with a focus on the assessment of the antimicrobial efficacy of pure nanohydroxyapatite, Cu^2+^-doped nanohydroxyapatite, ozonated olive oil-loaded nanohydroxyapatite, and Cu^2+^-doped nanohydroxyapatite, respectively. Their potential antimicrobial activity was investigated against *Streptococcus mutans, Lactobacillus rhamnosus*, and *Candida albicans*. Among all tested materials, the highest efficacy was observed in terms of ozonated olive oil. The studies were performed using an Ultraviolet–Visible spectrophotometry (UV-Vis), electron microscopy, and statistical methods, by determining the value of Colony-Forming Units (CFU/mL) and Minimal Inhibitory Concentration (MIC).

## 1. Introduction

Nowadays, the application of biomaterials is gaining popularity due to their high versatility. The development of modern medical science is based on the use of biomaterials, such as hydroxyapatite (HAp), to replace damaged hard tissue. Hydroxyapatite is the main inorganic component of bones and teeth, and it is related to the resorption and precipitation processes of calcium phosphates as well as the adsorption and formation of bones, dentine, and cementum [1]. Mainly, it crystallizes in the form of nanoplates or nanorods with an average size of approximately 50 nm × 25 nm × 2 nm [2,3]. The natural HAp is non-stoichiometric and poorly crystalline, and it contains numerous ionic substitutions, e.g., Mg^2+^, Na^+^, K^+^, Sr^2+^, Zn^2+^, Mn^2+^, Cu^2+^, Co_3_^2−^, F^−^, and Cl^−^ [1,4]. Its synthetic form is isostructural and chemically similar to bone apatite and possesses a strong affinity for ion exchange, which causes its high bioactivity [5]. Its biological properties are determined by such parameters as Ca/P molar ratio, the type of ionic dopants in the crystal lattice, or particle size and morphology. Stoichiometric HAp has a typical lattice structure described as (A_10_(BO_4_)_6_C_2_), where A, B, and C are defined by Ca^2+^, PO_4_^3−^, and OH^−^, respectively, with a calcium-to-phosphate ratio of 1.67 [6]. The HAp is non-immunogenic and non-toxic due to the outstanding bioactivity and biocompatibility. Moreover, synthetic hydroxyapatite has been widely applied as a bone substitute for the reconstruction of bone defects in maxillofacial surgery as well as orthopedics [1,7]. Furthermore, it can be used as a filler for repairing cavities on the enamel surface [8].

In orthopedics, bacterial adhesion on implant surfaces is the most predominant problem of post-surgical infections. Several studies have reported that the Ag^+^, Cu^2+^, and Zn^2+^ ions are essential for preventing or minimizing initial microorganism adhesion [9,10,11]. Among them, the Cu^2+^ ion occupies a prominent position as an antibacterial agent, because it reveals the highest inhibition of bacteria growth with simultaneous tolerable cytotoxicity for tissue cells, as was reported by Heidenau et al. [10]. The antimicrobial activity of cooper ions can be ascribed by several mechanisms. Under aerobic conditions, the Cu^2+^ ion is proposed to be catalyzed producing hydroxyl radicals via the Fenton and Haber–Weiss reactions [12]. The possible mechanisms of action between the Cu^2+^ ion-containing compounds and the microorganism are based on the structural damage of the cell membrane causing its permeability and finally cell death, the deactivation of proteins by binding metal ions, and the interaction with microbial nucleic acids preventing microbial replication [13,14]. In the presented study, it has been decided to choose 1 mol% Cu^2+^ due to the fact that copper-doped nanohydroxyapatite (nHAp) retains an antimicrobial effect even at low Cu^2+^ content, while its cytotoxicity against normal cells remains low [15]. Chui Ping Ooi et al. showed that the survival ratio of osteoblasts decreased as the Cu^2+^ content increased [16], while Nam et al. [17] confirmed, that Cu^2+^ concentration and contact time do not affect to the phase composition, but affect the crystal size and morphology. Moreover, from the physicochemical point of view, the crystal structure of the hydroxyapatite is stable at this (1 mol%) concentration of dopant. The presence of secondary phases could be observed with an increase of copper ions content in nHAp crystal lattice, as was presented by Sumathi Shanmugam et al. [13].

Cu^2+^ ions have a strong activity against fungi and bacteria [18]. Moreover, the bactericidal effect of metal ions as well as nanoparticles has been attributed to their small size and a high surface-to-volume ratio, allowing close interaction with microbial membranes. Nanoparticles have coated surfaces and can be useful in various medical fields e.g., as cements or coatings in surgery, antimicrobial dressings, and actively targeted biomaterials [19,20].

The use of ozone in dentistry has increased in recent years due to its high oxidative power stimulating the immune response and blood circulation, together with its strong antimicrobial activity [21]. It has been demonstrated to be useful in controlling the physiology of microorganisms in dental plaque [22]. Ozone works synergistically—inducing the modification of intracellular contents and damaging the cytoplasmic membrane of cells [23]. Some medical products such as Ozonosept (see Section 2: Materials and Methods) contain ozone. It is fabricated during the process of ozonation of olive oil. Ozone is kept in the form of stable chemical compounds—ozonides. The ozonides show antibacterial, antifungal, and antiviral activity [24]. The antimicrobial activity of ozonated olive oil is related to the Criegee Mechanism i.e., a slow release of peroxides [25]. When an ozonide contacts with tissue, then carbonyl oxide reacts with water, and hydroxyhydroperoxide is produced. According to the Metrum Cryoflex leaflet, the Ozonosept has confirmed antimicrobial properties against *Staphylococcus aureus, Pseudomonas aeruginosa, Escherichia coli, Propionibacterium acnes*, and *Candida albicans*.

This study aimed to evaluate the selected materials against *Candida albicans, Streptococcus mutans,* and *Lactobacillus rhamnosus*. This set of microorganisms was used in our previous paper, Wiglusz et al. [26]. According to the literature, *C. albicans* has strong adhesive properties. Moreover, *S. mutans* and *L. rhamnosus* are related to formation of a subgingival plaque. Moreover, these strains are referential in the case of in vitro studies for biomaterials.

The co-administration of nanoparticles and ozonated olive oil has not been extensively studied against microbial species isolated from persistent endodontic infections. Recent studies [27,28] have shown that the combination of ozonated olive oil and chitosan nanoparticles has a more significant killing effect—it prevents biofilm formation and eradicates resistant endodontic pathogens from root canals. The novelty of this work is its evaluation and comparison of the antimicrobial activity of the proposed materials on their own and in various compositions. Such a comparative study gives the opportunity to reveal the specificity of these materials toward various microorganisms including bacterial strains and pathogen yeast as well as ultimately leading to the better utilization of nanoparticles and ozonated olive oil.

## 2. Materials and Methods 

### 2.1. Synthesis of Nanocrystalline Hydroxyapatite

The studies were carried out on the following materials: (i) nHAp, (ii) nHAp doped with Cu^2+^ ions, (iii) nHAp with the addition of ozonated olive oil (Ozonosept, Metrum Cryoflex Sp. z o.o., Sp. K., Łomianki, Poland), and (iiii) nHAp doped with Cu^2+^ ions and loaded with ozonated olive oil. The amount of ozone in olive oil was 100 mg/mL, as proven by Magnetic Resonance Spectroscopy (600Mhz/16 tesla) (Bruker Corporation, Billerica, MA, USA) by the manufacturer. 

The nHAp nanocrystals of Ca_10_(PO_4_)_6_(OH)_2_ and Ca_9.9_Cu_0.1_(PO_4_)_6_(OH)_2_ were synthesized by the wet chemistry method at the Institute of Low Temperature and Structure Research, Wroclaw, Poland. Analytical grade Ca (NO_3_)_2_·4H_2_O (99.0%, Alfa Aesar, Haverhill, MA, USA), NH_4_H_2_PO_4_ (99.0%, Fluka^TM^, Honeywell Specialty Chemicals Seelze GmbH., Seelze, Germany), and Cu (NO_3_)_2_∙2.5H_2_O (98.0–102.0%, Alfa Aesar) were used as the starting materials. The pH was regulated by NH_3_∙H_2_O (99%, Avantor Performance Materials Poland S.A., Gliwice, Poland). The concentration of Cu^2+^ ions was 1 mol% to the overall molar content of calcium cations. All substrates were dissolved and mixed. The pH of the reaction mixture was adjusted to 10 with an ammonia solution. The reaction was performed at 100 °C for 60 min. The obtained product was washed several times with deionized water and dried at 70 °C for 24 h. The final product was heat-treated at 400 °C for 3 h.

### 2.2. Characterisation 

The apatite crystal structure was confirmed by the X-ray diffraction technique (XRD). The XRD patterns were measured (five times for each samples) by using a PANalytical X’Pert Pro X-ray diffractometer (Malvern Panalytical Ltd., Malvern, UK) equipped with Ni-filtered Cu Kα1 radiation (Kα1 = 1.54060 Å, U = 40 kV, I = 30 mA). The measurements were done in the range of 3–70° (2*θ*). The Thermo Fisher Scientific Nicolet iS50 FT-IR spectrometer (Waltham, MA, USA) equipped with an Automated Beamsplitter exchange system (iS50 ABX containing DLaTGSKBr detector), which had a built-in all-reflective diamond Attenuated Total Reflectance (ATR) module (iS50 ATR), Thermo Scientific Polaris™ and HeNe laser, was used to record the FT-IR spectra (five times for each samples). The Fourier Transform Infrared (FT-IR) spectra in the mid-IR region (4000–400 cm^−1^) were measured using the standard KBr pellet method, while in the case of the far-IR region (400–100 cm^−1^), a Nujol suspension was used. Raman measurements (five times for each sample) were carried out with a Micro-Raman system Renishaw InVia Raman spectrometer equipped with a confocal DM 2500 Leica optical microscope (Wotton-under-Edge, Gloucestershire, UK), a thermoelectrically cooled Charge-Coupled Device CCD was used as a detector of the Raman spectra recorded. An argon laser operating at 831 nm was used. The chemical composition was performed by using an FEI Nova NanoSEM 230 scanning electron microscope (SEM, Hillsboro, OR, USA) with an energy-dispersive X-ray spectrometer (EDAX Genesis XM4). The Scanning Electron Microscopy coupled with Energy Dispersive X-ray Spectroscopy (SEM-EDS) was used for qualitative and quantitative analysis of materials. The spectra were recorded three times for each sample, and the calculated value is an average result.

The tests were carried out on reference strains *Streptococcus mutans* (ATCC 25175), *Lactobacillus rhamnosus* (ATCC 9595), and *Candida albicans* (ATCC 90028).

The aim of the study was to conduct preliminary studies associated with the activity of the tested compounds against different microorganisms in their planktonic forms. The next stage will be related to an evaluation of the activity of the selected compounds against a mature structure of microbial biofilms.

### 2.3. Spectrophotometric Examination 

A suspension of 0.5 McFarland density (1.5 × 10^8^ CFU/mL in case of bacteria and 1.5 × 10^6^ CFU/mL in case of fungi) in liquid medium Sabouraud Broth (Biomaxima) Brain Heart Infusion (BHI) Broth (Biomaxima) and De Man, Rogosa and Sharpe MRS Broth (Biomaxima) were prepared from fresh culture of the analyzed strains for *Candida albicans, Streptococcus mutans*, and *Lactobacillus rhamnosus*, respectively. First, 1 mL of the suspension prepared in this way was incubated with nHAp at a concentration of 0.1% and 1% (both pure and with an admixture of Cu^2+^ and ozonated olive oil). According to previous studies including substituted hydroxyapatites with antibacterial properties it has been decided to choose two different concentrations of nHAp (0.1% and 1%) [15,16,17,28,29]. The samples were incubated at 37 °C (aerobic, anaerobic (GENbag anaer, Biomerieux), and microaerophilic (GENbag microaer, Biomerieux, Warsaw, Poland)) for 4 h and 24 h with shaking. After the incubation period, 100 µL of the suspension was transferred to the appropriate well of a 96-well plate according to the following scheme:

- Growth control (1 mL suspension of microorganisms);
- Sterility control (1 mL medium);
- Compound control (1 mL medium and 0.1% and 1% concentration);
- Test sample (1 mL of microorganism suspension and analyzed compound at a concentration of 0.1% and 1%).

The reading was made on a Biochrom Asys UVM 340 spectrophotometer at 595 nm (Biochrom Ltd., Holliston, MA, USA).

### 2.4. Determining the Value of the Colony-Forming Units, CFU/mL

Suspensions of 0.5 McFarland density (1.5 × 10^8^ CFU/mL in case of bacteria and 1.5 × 10^6^ CFU/mL in case of fungi) in liquid Sabouraud, BHI, and MRS medium were prepared from fresh culture of the analyzed strains for *C. albicans*, *S. mutans*, and *L. rhamnosus*, respectively. In this way, 1 mL of the prepared suspension was incubated with nHAp at concentrations of 0.1% and 1% (pure, as well as doped with Cu^2+^ ions and an addition of ozonated olive oil). The samples were incubated at 37 °C (aerobic, anaerobic (GENbag anaer, Biomerieux, Warsaw, Poland), and microaerophilic (GENbag microaer, Biomerieux)) for 24 h with shaking. After the incubation period, 100 μL of the suspension was withdrawn, and a series of dilutions were made in geometric progress (10^−1^–10^−6^). After plating on a solid medium (appropriate for the strain), the plates were incubated; then, the grown colonies were counted, and the value of the colony-forming units (CFU/mL) was determined. All tested samples were subjected to triplicate procedure.

Together with the test sample, a control test was done, which was a suspension of the analyzed strain. The antimicrobial properties of ozonated olive oil have also been evaluated. The proportions of the ozonated olive oil together with microbial strain were 1 mL of bacterial/fungal suspension and 1 mL of ozonated olive oil.

### 2.5. Determining the Value of the Minimal Inhibitory Concentration, MIC

First, 100 mL of the medium (appropriate for the strain) was applied to the wells of the 96-well plate. Then, 100 µL of the nHAp suspension (pure and doped with Cu^2+^ ions and an addition of ozonated olive oil) was applied to the appropriate plate rows and diluted geometrically to a concentration range of 9.7–5000 µg/mL. After adding 20 µL of the diluted microorganism culture, the plate was incubated (37 °C; aerobic, anaerobic and microaerophilic). After the incubation period, the minimum inhibitory concentration value was read visually.

### 2.6. Scanning Electron Microscopy

Suspensions of 0.5 McFarland density (1.5 × 10^8^ CFU/mL in case of bacteria and 1.5 × 10^6^ CFU/mL in case of fungi) in liquid Sabouraud, BHI, and MRS medium were prepared from a fresh culture of the analyzed strains for *C. albicans*, *S.mutans*, and *L. rhamnosus*, respectively. First, 1 mL of the suspension prepared in this way was incubated with nHAp at a concentration of 0.1% and 1% (pure HAp as well as doped with Cu^2+^ ions and the addition of ozonated olive oil). The samples were incubated at 37 °C (aerobic, anaerobic (GENbag anaer, Biomerieux), and microaerophilic (GENbag microaer, Biomerieux)) for 24 h with shaking. After the incubation period, 100 μL of the suspension was transferred to the appropriate well of a 12-well plate, fixed, sprayed with gold, and evaluated in a ZEISS scanning electron microscope model EVO LS15 (Carl Zeiss, Oberkochen, Germany).

### 2.7. Statistical Methods

For all quantitative features (number of colony-forming units, CFU/mL), their distribution was checked for compliance with the normal distribution. The conformity assessment was carried out with the Shapiro–Wilk test.

Qualitative variables (strains of microorganisms) are presented in the abundance tables (contingency) in the form of abundance (*n*) and proportion (%). The chi-squared test was used to assess the strength of the relationship between the two variables. In cases where the number expected in one of the tables (2 × 2) was less than 5, the Fisher’s exact test was used. Mean values (±M) and standard deviations (±SD) were calculated for all measurable features. The homogeneity of variance was checked by the Bartlett and Levene test.

Analysis of variance (Anova) was used to compare the averages in several groups. Whether the analyzed feature in each of the examined groups had normal distribution and equal variances had been checked earlier. If the probability corresponding to the value of the Snedecor F distribution was lower than the assumed level of significance (*p* < 0.05), then multiple comparison tests (post hoc) were performed to determine which group significantly differs from the others. The Tukey test was used for this purpose.

The Statistica version 12.5 program (StatSoft, Tulsa, OK, USA) was applied for calculations and making charts.

## 3. Results

### 3.1. Structural Analysis

**Figure 1:**
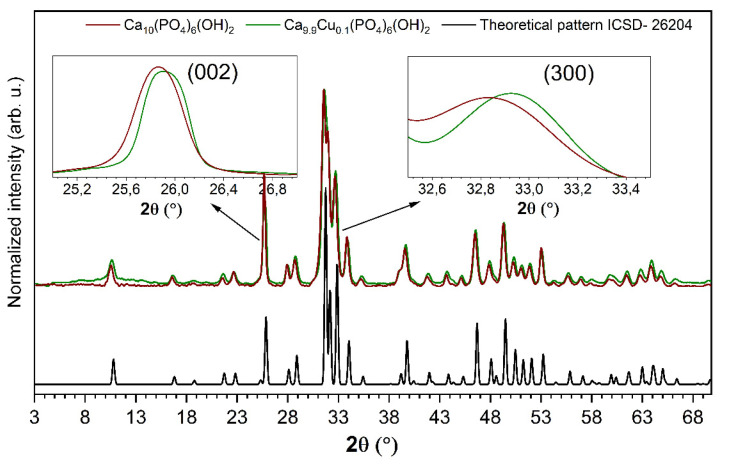
X-ray diffraction patterns of pure nanohydroxyapatite (nHAp) and nHAp doped with 1 mol% Cu^2+^ after heat treatment at 400 °C with the indication of (002) and (300) planes shift induced by doping with Cu^2+^ ions.

Structural analysis of the Ca_10_(PO_4_)_6_(OH)_2_ nanocrystals as well as nHAp doped with 1 mol% Cu^2+^ ions was performed by using the XRD technique. The X-ray diffraction patterns of pure and Cu^2+^-doped nHAp are presented in Figure 1. As it can be seen, the observed XRD patterns are in good agreement with the reference hexagonal phase of nHAp (no. ICSD-26204) ascribed to the P6_3_/m space group [30]. The successful replacement of calcium ions by cooper ions in the crystal structure was confirmed—no additional peaks originating from other phases were observed. The efficient substitution of Ca^2+^ ions by Cu^2+^ ions has been clearly confirmed by shifting the positions of the diffraction peaks. Hydroxyapatite doped with Cu^2+^ ions exhibits a slight shift in the position of the (002) plane (c-plane) and (300) plane (a-plane), [13,28,31]. A shift toward higher 2*θ* angles was related to the decrease in the cell parameters induced by the substitution of the bigger Ca^2+^ cation (CN_9_ = 1.18 Å, Ca^2+^ CN_7_ = 1.06 Å, where CN is coordination number) by the smaller Cu^2+^ cation (CN_7_ = 0.73 Å) [28,32,33]. 

Moreover, the X-ray diffraction can be used to determine the presence of the OCP (octacalcium phosphate) phase. The OCP structure can be described as an alternative stacking of “apatite” layers and “hydrated” layers [34]. Most of the OCP reflections within the 2*θ* range of 10–60° overlapped with those belonging to the hydroxyapatite structure. However, three reflections are specific to OCP in the low 2*θ* range of 4–10°, at 4.7°, 9.5°, and 9.8° with the relative intensities equal to 100%, 8%, and 8%, respectively [35]. In the case of the studied materials, Bragg peaks at very low 2*θ* angles, especially the most intense (100) line, were not observed. Meanwhile, the most characteristic diffraction peaks belonging to the hydroxyapatite structure were found at 2*θ* equal to 31.8°, 32.2°, 32.9°, and 25.9°. 

### 3.2. Infrared Spectra

**Figure 2:**
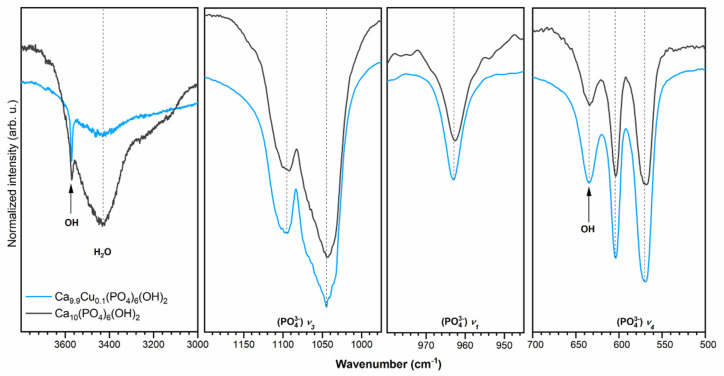
FT-IR spectra of nHAp and nHAp doped with 1 mol% Cu^2+^ with an indication of typical active vibrational bands.

The infrared spectra of investigated materials are shown in Figure 2. The absorption bands have been ascribed based on literature data [36,37,38]. The peaks at 1045 cm^−1^ and 1095 cm^−1^ correspond to the antisymmetric triply degenerate stretching vibrations of phosphate groups (PO_4_^3−^)ν_3_. The peak at 963 cm^−1^ belongs to the symmetric non-degenerate stretching vibrations of phosphate groups (PO_4_^3−^ν_1_), while the modes at 604 cm^−1^ and 570 cm^−1^ identify the triply degenerate vibration (PO_4_^3−^)ν_4_. The presence of the absorption band at 634 cm^−1^, belonging to the librational mode of the –OH group, clearly indicates the nHAp structure. The peak observed at 3571 cm^−1^ is related to the stretching mode of the –OH group. The broad absorption band with a maximum at 3430 cm^−1^ corresponds to the typical vibrations of water molecules.

**Figure 3:**
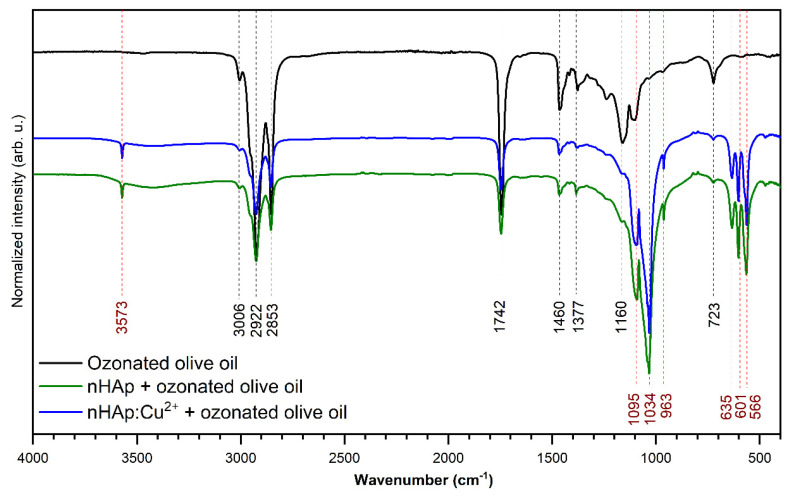
FT-IR spectra of ozonated olive oil as well as nHAp and nHAp doped with 1 mol% Cu^2+^ and loaded with ozonated olive oil.

The ATR-IR absorption spectra (in the range of 4000–400 cm^−1^) of the ozonated olive oil and nHAp with the addition of ozonated olive oil as well as nHAp doped with Cu^2+^ and loaded with ozonated olive oil are presented in Figure 3. The bands related to ozonated olive oil and hydroxyapatite have been marked with black and red dashed lines, respectively. According to the spectroscopic results, it has been proven that the ozonated olive oil was adsorbed on the obtained compounds. The most typical peak associated with ozonated olive oil, indicating the existence of an ozonide ion, is located at 1104 cm^−1^ and is correlated with the ozonide CO stretching mode [39]. The peak at 3006 cm^−1^ is associated with the C–H stretching vibration of the cis double bond. There are also two intense peaks at 2922 and 2853 cm^−1^, which correspond to the C–H asymmetric stretching vibrations of both –CH_2_ and –CH_3_ groups. The peak at 1742 cm^−1^ demonstrates C=O vibrations. The modes around 1460 and 723 cm^−1^ are assigned to the bending C–H vibration, while the peak at 1160 cm^−1^ is attributed to the C–O bands [40].

### 3.3. Micro-Raman Spectra

**Figure 4:**
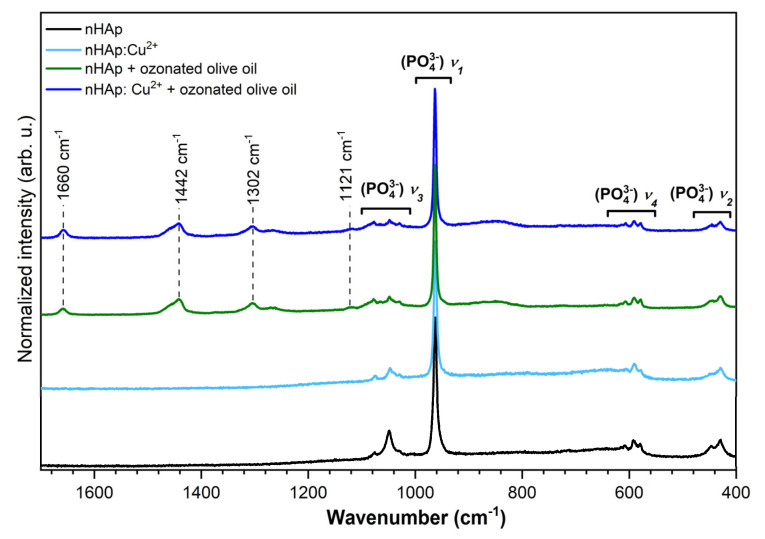
The micro-Raman spectra of nHAp and nHAp doped with 1 mol% Cu^2+^ as well as both materials loaded with ozonated olive oil.

The micro-Raman spectra of pure and Cu^2+^-doped nHAp were recorded and presented in Figure 4. The spectra of the Cu^2+^-doped nHAp contain four characteristic vibrational transitions of phosphate groups. The maximum of the most intense peak is located at 961 cm^−1^ and is correlated with the symmetric stretching mode of the phosphate groups (PO_4_^3−^)ν_1_. The three overlapping vibration modes at 1075, 1046, and 1028 cm^−1^ are attributed to the asymmetric stretching of (PO_4_^3−^)ν_3_. In the region of the (PO_4_^3−^)ν_2_ bending mode, there are two peaks at 592 and 580 cm^−1^. The positions of 452 and 430 cm^−1^ are associated with (PO_4_^3−^)ν_4_ bending modes. The analysis of the Raman spectrum related to pure nHAp revealed one distinguishing peak at 961 cm^−1^ associated with (PO_4_^3−^)ν_1_ vibration. Moreover, the peaks correlated with the ν_2_, ν_3_, and ν_4_ vibrational transitions of phosphate groups were not clearly detected [16,19]. The spectra of the samples loaded with ozonated olive oil reveal additional bands at 1660 cm^−^^1^ derived from aliphatic unsaturation, at 1442 cm^−^^1^ associated with the deformation of the CH_2_ group, and at 1302 cm^−^^1^ correlated to the twisting of the CH_2_ group [41].

### 3.4. EDS Analysis

**Figure 5:**
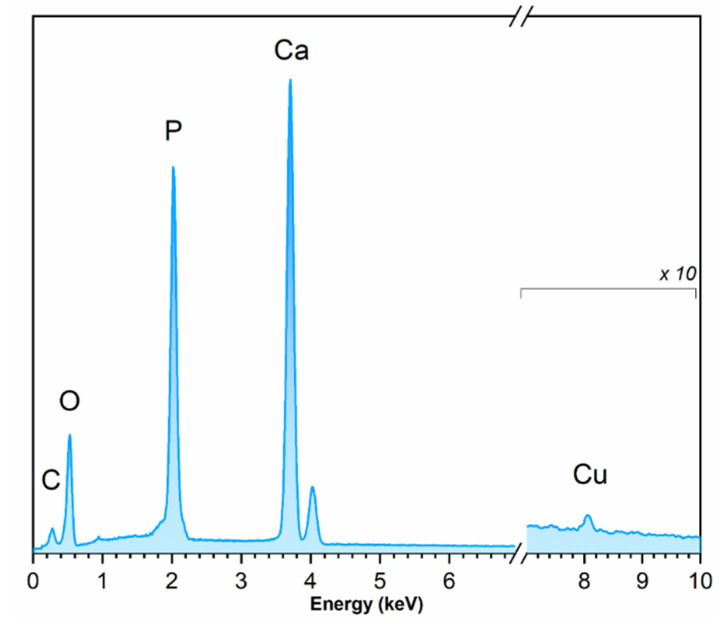
EDS spectrum of nHAp doped with 1 mol% Cu^2+^ after heat treatment at 400 °C.

The EDS spectra (Figure 5) recorded for the samples was applied to identify and quantify the elements in the nHAp:Cu^2+^. The resulting contents of Ca, Cu, and P in the studied material were 28.2 at%, 0.39 at%, and 18.1 at%, respectively. The calculated value of the (n_Ca_+n_Cu_)/n_P_ ratio was equal to 1.5—close to the theoretical n_Ca_/n_P_ ratio. The calculated concentration of Cu^2+^ ions was equal to 0.1 mol%, which stays in agreement with the theoretical value. 

### 3.5. Spectrophotometric Indication

The range of reduction of viable *C. albicans* cells after 4 h incubation under modified nHAp ranged from 46 to 87% for a concentration of 0.1% and 44 to 84% for a concentration of 1%. In the case of *S. mutans* and *L. rhamnosus*, it was respectively 47–97% and 77–100%, as well as 38–52% and 24–54% for concentrations of 0.1% and 1%.

After 24 h incubation, the degree of reduction was in the range of 21–89% and 50–52% in the case of *C. albicans*, and 41–75% and 19–81% for *S. mutans*. In the case of *L. rhamnosus*, these values ranged from 11 to 62% and 5 to 88% for 0.1% and 1%, respectively. The results are shown in Tables S1 and S2. 

### 3.6. Determining the Value of the Minimal Inhibitory Concentration

The Minimum Inhibitory Concentration (MIC) value of undoped and Cu^2+^-doped nHAp loaded with ozonated olive oil against *C. albicans* was > 5000 and 5000 µg/mL, respectively. In the case of *S. mutans* MIC, undoped and Cu^2+^-doped nHAp was > 5000 µg/mL, and in the case of Cu^2+^-doped nHAp loaded with ozonated olive oil, it reached 5000 µg/mL. In the case of *L. rhamnosus*, the value of the minimum growth inhibitory concentration for all analyzed compounds exceeded the value of 5000 µg/mL. The results are shown in Table 1.

### 3.7. Determining the Value of Colony-Forming Units, CFU/mL

**Figure 6:**
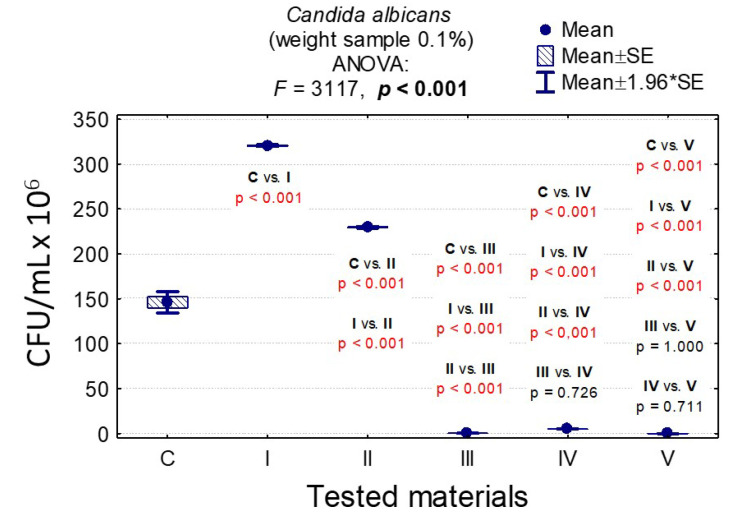
Average Colony-Forming Units (CFU)/mL values and standard deviation (M ± SD) for *Candida albicans* after contact with tested materials (C—control group, I—nHAp, II—Cu^2+^-doped nHAp, III—nHAp with the addition of ozonated olive oil, IV—nHAp doped with Cu^2+^ and loaded with ozonated olive oil, V—ozonated olive oil) for weights of 0.1% and results of the analysis of variance (ANOVA) and multiple comparisons by a post-hoc test. Incubation took 24 h.

**Figure 7:**
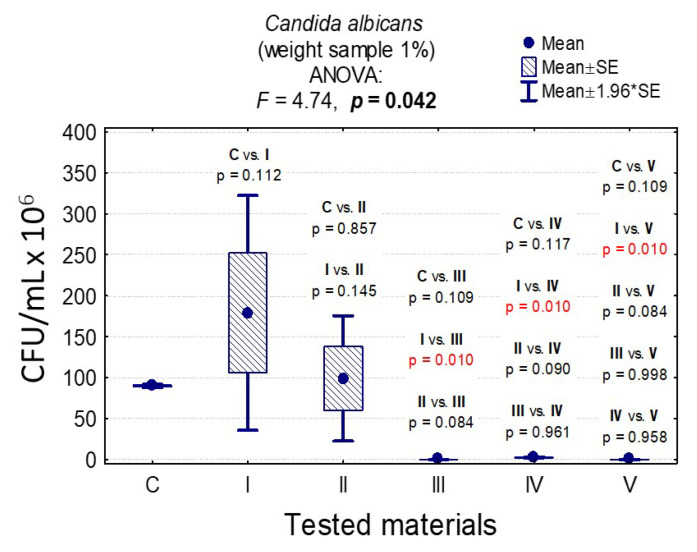
Average CFU/mL values and standard deviation (M ± SD) for *Candida albicans* after contact with the tested materials (C—growth control, I—nHAp, II—Cu^2+^-doped nHAp, III—nHAp with the addition of ozonated olive oil, IV—nHAp doped with Cu^2+^ and loaded with ozonated olive oil, V—ozonated olive oil) for weights of 1% and results of the analysis of variance (ANOVA) and multiple comparisons by a post-hoc test. Incubation took 24 h.

**Figure 8:**
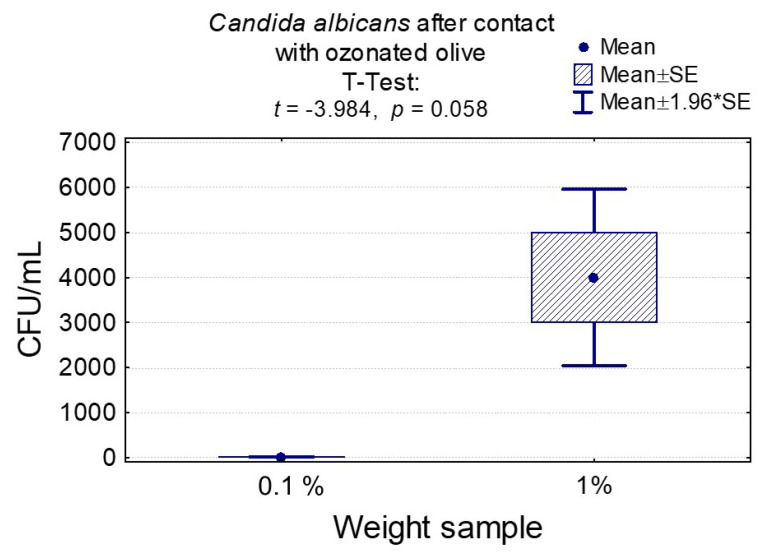
Average CFU/mL value and standard deviation (M ± SD) for *Candida albicans* after temporary contact with olive ozone in groups differing in weight and significance of test result. Incubation took 24 h.

**Figure 9:**
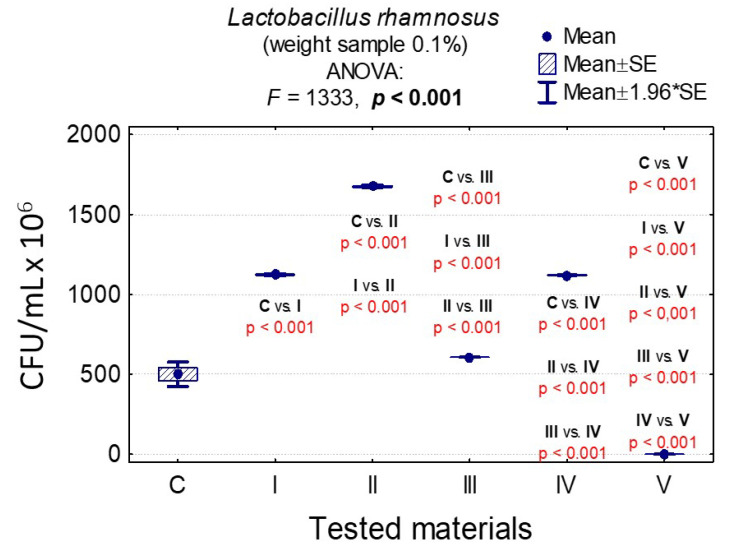
Average CFU/mL values and standard deviation (M ± SD) for *Lactobacillus rhamnosus* after temporary contact with the tested materials (C—growth control, I—nHAp, II—Cu^2+^-doped nHAp, III—nHAp with the addition of ozonated olive oil, IV—nHAp doped with Cu^2+^ and loaded with ozonated olive oil, V—ozonated olive oil) for weights of 0.1% and results of analysis of variance (ANOVA) and multiple comparisons by a post-hoc test. Incubation took 24 h.

**Figure 10:**
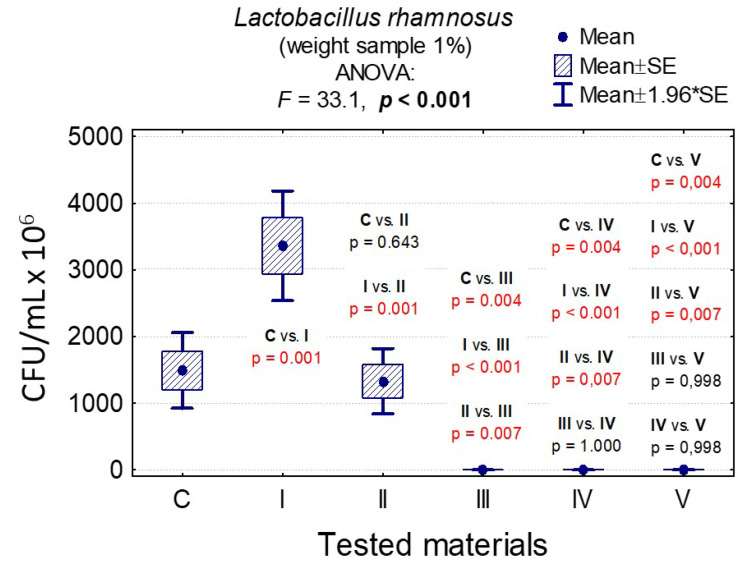
Average CFU/mL values and standard deviation (M ± SD) for *Lactobacillus rhamnosus* after temporary contact with the tested materials (C—growth control, I—nHAp, II—Cu^2+^-doped nHAp, III—nHAp with the addition of ozonated olive oil, IV—nHAp doped with Cu^2+^ and loaded with ozonated olive oil, V—ozonated olive oil) for weights of 1% and results of analysis of variance (ANOVA) and multiple comparisons by a post-hoc test. Incubation took 24 h.

**Figure 11:**
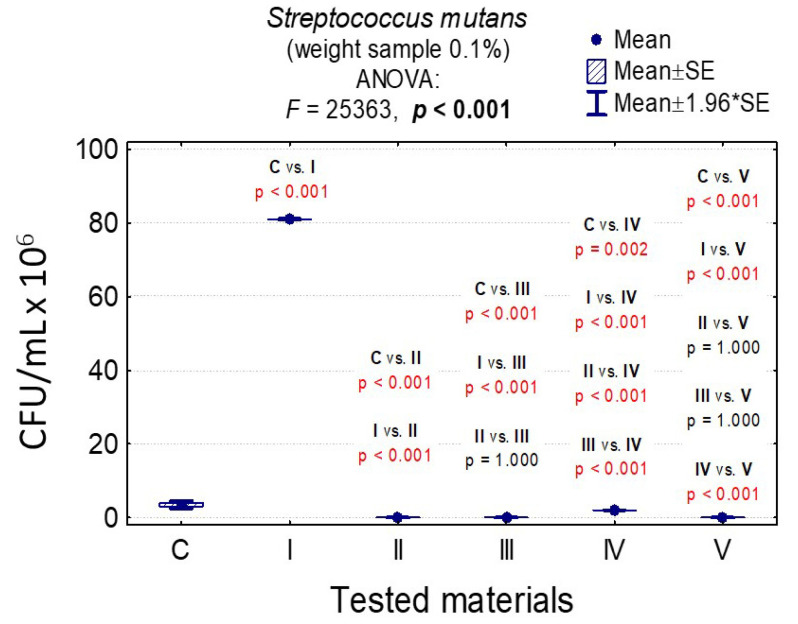
Average CFU/mL values and standard deviation (M ± SD) for *Streptococcus mutans* after temporary contact with the tested materials (C—growth control, I—nHAp, II—Cu^2+^-doped nHAp, III—nHAp with the addition of ozonated olive oil, IV—nHAp doped with Cu^2+^ ions and loaded with ozonated olive oil, V—ozonated olive oil) for weights of 0.1% and results of the analysis of variance (ANOVA) and multiple comparisons by a post-hoc test. Incubation took 24 h.

**Figure 12:**
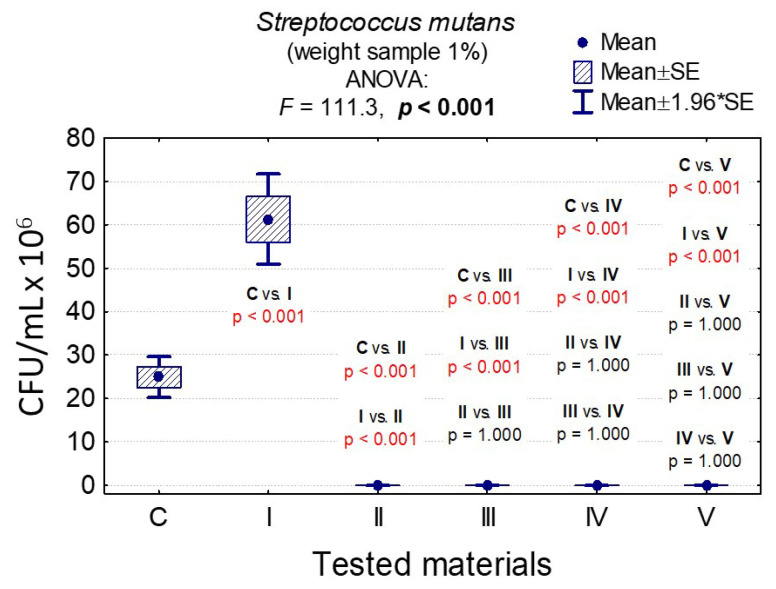
Average CFU/mL values and standard deviation (M ± SD) for *Streptococcus mutans* after temporary contact with the tested materials (C—growth control, I—nHAp, II—Cu^2+^-doped nHAp, III—nHAp with the addition of ozonated olive oil, IV—nHAp doped with Cu^2+^ and loaded with ozonated olive oil, V—ozonated olive oil) for weights of 1% and results of the analysis of variance (ANOVA) and multiple comparisons by a post-hoc test. Incubation took 24 h.

In the case of *C. albicans*, the range of Colony-Forming Units was 1 × 10^5^–3.20 × 10^8^ for 0.1% and 1.4 × 10^5^–1.79 × 10^8^ for 1%. In the case of *L. rhamnosus* and *S. mutans*, the range was 6.07 × 10^8^–1.67 × 10^9^, 1 × 10^3^–3.36 × 10^9^, 0–8.10 × 10^7^, and 0–6.13 × 10^7^, respectively. The results are presented in Tables S3–S5 and Figure 6, Figure 7, Figure 8, Figure 9, Figure 10, Figure 11 and Figure 12. It has been shown that the HAp doped with Cu^2+^ ions and the ozonated olive oil-loaded HAp doped with Cu^2+^ ions have caused a readable reduction of CFU/mL for *Candida albicans* and *Lactobacillus rhamnosus.* Moreover, the highest value of CFU/mL has been observed when pure nHAp was used against the *Candida albicans* as well as Cu^2+^ ions-doped HAp against *Lactobacillus rhamnosus.* Furthermore, the highest CFU/mL value has also been observed in the case of pure nHAp, while the lowest occurred with both nHAp with the addition of ozonated olive oil and nHAp doped with Cu^2+^ ions and loaded with ozonated olive oil against the *Streptococcus mutans* strain. On the other hand, the CFU/mL had been evaluated by the lowest values in contact with 1 mol% Cu^2+^-doped nHAp, 1 mol% Cu^2+^-doped nHAp with the addition of ozonated olive oil, and 1 mol% Cu^2+^-doped nHAp loaded with ozonated olive oil against *Streptococcus mutans* and *Candida albicans*. 

In the case of a sample with 0.1% nHAp, we observed statistically significant differences in the growth of the *C. albicans* colony for the growth control and all other materials (*p* < 0.001), pure nHAp (material I) and other materials (*p* < 0.001), and nHAp doped with Cu^2+^ ions and other materials (*p* < 0.001). The differences between materials containing ozonated olive oil (materials III, IV and V) turned out to be insignificant (*p* > 0.05); see Table S3 and Figure 6.

In the case of a sample weight of 1% nHAp, it was observed that there were significant statistical differences in the growth of *C. albicans* for pure nHAp (material I) and materials containing ozonated olive oil (materials III, IV, V); see Table S4 and Figure 7.

Ozonated olive oil turned out to be the best material for *C. albicans* (smallest colony growth). After two measurements, the sample weight did not have a significant statistical impact on the colony concentration (Tables S3–S5 and Figure 8).

In the case of sample weights of 0.1% nHAp, it was shown that there were statistically significant differences in the growth of *L. rhamnosus* colonies for all materials except pure nHAp and nHAp doped with Cu^2+^ ions and loaded with ozonated olive oil (*p* = 0.900). It was proven that the ozone olive has been the best material (Table S3 and Figure 9).

In the case of sample weights of 1% nHAp, it was observed that there were statistically significant differences in the growth of *L. rhamnosus* colonies for all materials except those containing ozonated olive oil (III, IV, and V), confirming that the ozonated olive oil has been the best material (Table S4 and Figure 10).

In the case of a weight sample of 0.1% nHAp, the slight increase of *S. mutans* colonies under the influence of Cu^2+^-doped nHAp, nHAp with the addition of ozonated olive oil and pure ozonated olive oil was observed, and the differences between them were insignificant (*p* = 1.000); see Table S3 and Figure 11.

In the case of a weight sample of 1% nHAp, the slight growth of *S. mutans* colonies under the influence of Cu^2+^-doped nHAp, nHAp with the addition of ozonated olive oil, as well as nHAp doped with Cu^2+^ and loaded with the addition of ozonated olive oil and pure ozonated olive oil was observed, and differences between them were insignificant (*p* = 1.000); see Table S4 and Figure 12. 

In all cases, the results of the variance analysis were statistically significant (for example, in Figure 12: *F* = 111.3 and *p* < 0.001. It means that the difference between at least one of the pairs of materials was significant). Moreover, the post-hoc tests were carried out Least Significant Difference (LSD) showing significant differences between the pairs of materials presented in Figure 12 (*p* < 0.001).

The difference between growth control and nHAp was significant (*p* < 0.001), and that was not the case in comparison with Cu^2+^-doped nHAp and nHAp with the addition of ozonated olive oil (*p* = 1.000).

In the case of a sample with a concentration of 0.1% nHAp, statistically significant differences were observed in the growth of *L. rhamnosus* colonies for all materials, except for nHAp and nHAp doped with Cu^2+^ ions and loaded with ozonated olive oil (*p* = 0.900). Moreover, regarding the sample weights of 1.0% nHAp, it was observed that there were statistically significant differences in the growth of *L. rhamnosus* colonies for all materials, except for those containing ozonated olive oil (III, IV, and IV). Furthermore, for the weight sample of 0.1% nHAp, the slight increase of *S. mutans* colonies under influence of Cu^2+^-doped nHAp, nHAp with the addition of the ozonated olive oil, and the pure ozonated olive oil was shown, and the differences between those materials were insignificant (*p* = 1.000).

Additionally, for the sample with a concentration of 1% nHAp, the growth of *S. mutans* colonies under the influence of materials II, III, IV, and V was the smallest, and the differences between those materials were insignificant (*p* = 1.000).

Taking into account the above, the result of the analysis of variance was statistically significant in all cases (*F* = 111.2 and *p* < 0.001; meaning that the difference was significant at least between one of the pairs of materials). Therefore, post-hoc tests were carried out (LSD) with the purpose of showing between which pairs of materials the differences were significant. For example, the difference between growth control and nHAp was significant (*p* < 0.001), but that was not the case in comparison with Cu^2+^-doped nHAp and nHAp with the addition of ozonated olive oil (*p* = 1.000); see Tables S6–S8.

### 3.8. Scanning Electron Microscopy 

**Figure 13:**
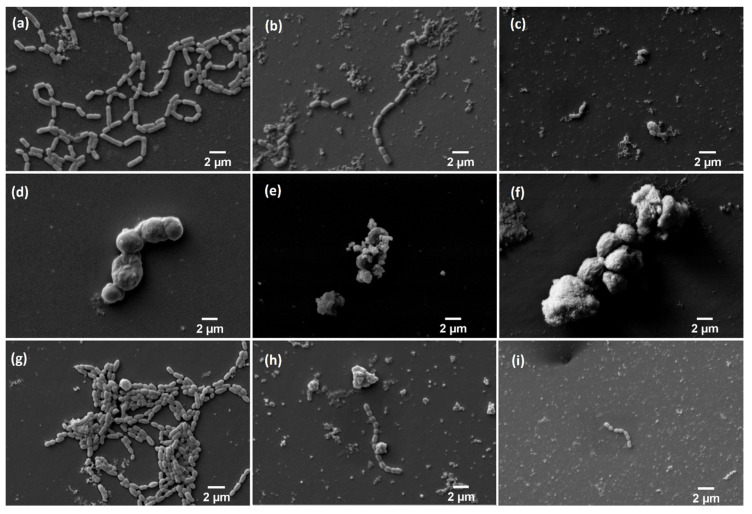
Scanning electron microscopy: (**a**) Growth control of *Lactobacillus rhamnosus*; (**b**) *Lactobacillus rhamnosus* with nHAp; (**c**) *Lactobacillus rhamnosus* with Cu^2+^-doped nHAp; (**d**) Growth control of *Candida albicans*; (**e**) *Candida albicans* with nHAp; (**f**) *Candida albicans* with Cu^2+^-doped nHAp; (**g**) Growth control of *Streptococcus mutans*; (**h**) *Streptococcus mutans* with nHAp; (**i**) *Streptococcus mutans* with Cu^2+^-doped nHAp; Mag = 10,000×.

In all cases, growth control is more abundant than when microorganisms are in contact with nHAp and Cu^2+^-doped nHAp (Figure 13). The action of nHAp doped with Cu^2+^ ions definitely affects the reduction of the number of bacteria (*Lactobacillus rhamnosus*, *Streptococcus mutans*). Changes are visible on *Candida albicans* surface—it is not smooth and oval, but more angular instead. Additionally, it looks as if it was dehydrated. 

## 4. Discussion

Hydroxyapatite is an inorganic component of bones and teeth, acting as a scaffold and giving them mechanical properties. In addition, it stimulates bone development in small bone defects and can be used as a coating material for implants [42]. In a medium containing the nHAp particle, bacteria may adhere to the solid and co-aggregate. It has been reported that biofilms that could be formed at 15 min after inoculation on nHAp disks consist mainly of single, non-aggregated cells [43].

The relationship between the size of nHAp and bacterial adhesion is crucial because of an effect on slower plaque formation. The nHAp scale allows having enhanced physical and chemical properties, including increased wettability, roughness, and adsorption of proteins [44]. Non-aggregated and condensed nHAp particles adsorb on bacterial surfaces in vitro [45]. They interact with bacteria and thus reduce their adhesion. Severin AV et al. [46] investigated the interaction of the nHAp nanocrystals with *Staphylococcus aureus* bacteria. Moreover, the nHAp nanocrystallites adhere to the surface of bacteria, significantly reducing their ability to form colonies.

The activity of nHAp against the planktonic form of the chosne microorganisms has been evaluated in this study. The degree of reduction of viable cells by pure nHAp (% viable microorganisms) was minimal after using pure nHAp. However, it is worth noting that with increasing exposure time as well as a concentration of pure nHAp (material I), the reduction of *C.* *albicans* initially increased at a concentration of 0.1% nHAp from 1% after 4 h to 14% after 24 h and at a concentration of 1% nHAp from 9% after 4 h to 37% after 24 h. In the case of other microorganisms, no relationship was found. Whereas, after doping or mixing nHAp with other reagents, a significant difference in the reduction of cells viability was observed in groups of nHAp doped with Cu^2+^ ions, loaded with ozonated olive oil, or both. After 4 hours incubation, the reduction range of viable *C.* *albicans* cells for pure nHAp was 1% at a concentration of 0.1% and 9% for a concentration of 1%.

The nHAp appeared to enhance biofilm formation by increasing glucosyltransferase transcription, which resulted in an increase in the production of insoluble glucans. Since the demineralization of nHAp in enamel caused by acids, including those produced by bacteria in the plaque, is important in the development of dental caries, nHAp is used in toothpastes for the remineralization of enamel [47]. In the current study, we have examined the effect of nHAp on the growth of *S.* *mutans* in two different media and a nutrient-rich environment. While exploring the extinction value measured as the percentage of cell reduction, it is observed that the antimicrobial activity of nHAp combined with Cu^2+^ ions is higher than that of the pure nHAp, although it shows less efficacy than nHAp with the addition of ozonated olive oil.

The nHAp doped with Cu^2+^ ions is distinct from other materials such as nHAp, nHAp with ozonated olive oil, as well as nHAp with Cu^2+^ ions with ozonated olive oil and ozone olive alone, for the reason that it is more efficient after 4 hours than after 24 h in both concentrations of nHAp (0.1% and 1%), unlike the other materials. It significantly reduced microorganism growth.

With regard to nHAp containing Cu^2+^ ions and ozonated olive oil, which is nHAp doped with Cu^2+^ ions and loaded with olive oil, it is apparently more effective than other materials in reducing the number of microorganisms. The only exception is *L.* *rhamnosus* after 4 h incubation, compared to nHAp with ozonated olive oil. nHAp at a concentration of 0.1% is the most efficient in the reduction of *S.* *mutans* in nHAp doped with Cu^2+^ ions, similarly to nHAp with ozonated olive oil. A similar effect is obtained regarding nHAp doped with Cu^2+^ ions, where the lack of growth can be observed. Once again, the highest efficiency was detected in the case of *S. mutans*, which is the same in materials with ozonated olive oil. nHAp doped with Cu^2+^ ions caused a statistically insignificant reduction of growth in the *L. rhamnosus* colony and *C. albicans.* Other studies also prove the antimicrobial properties of Cu^2+^ ions [48,49]. 

Studies prove a wide spectrum of activity that increases the field of application of nHAp doped with Cu^2+^ ions. It is suggested that it would have a wide spectrum of future use, for instance, in orthopaedics and bone prosthesis or dentistry and teeth implants [13,50].

Virgin olive oil has an abundance of unsaturated fatty acids due to an especially high content of oleic acid, which is a monounsaturated omega−9 (*n*−9) fatty acid. The process of ozonization oxides unsaturated bonds with the simultaneous formation of peroxidic substances, which results in the higher antifungal and antibacterial potential of ozonated substances [51].

The ozonated olive oil oil and its antimicrobial properties had been tested in some clinical trials concerning different diseases, for instance, in the gynecological or dentistry field. In terms of vulvovaginal candidiasis—that is an inflammation of vagina often caused by *Candida* species such as *Candida albicans* or NCAC (*non-Candida albicans Candida* species) such as *Candida glabrata, Candida krusei, Candida parapsilosis, Candida tropicalis*—it was found that the ozonated olive oil is equally effective as clotrimazole in a significant reduction of symptoms. Moreover, it also led to a negative culture growth, and there were no significant differences in terms of reducing itching and leukorrhea, although the burning sensation was diminished more effectively by clotrimazole [52].

Other studies provide an evaluation of ozonated olive oil in the treatment of chronic periodontitis, which is a disease caused mainly by bacteria, resulting in an inflammatory process of gums and tissues of the oral cavity. Although the clinical study was limited in terms of patient numbers, it was found that ozonated olive oil was effective not only as an adjunct to scaling and root planning but also in monotherapy. Nevertheless, when used as an adjunctive therapy, the patients’ dentinal hypersensitivity had significantly risen [53]. Those findings suggest a high level of efficacy of ozonated olive oil as an antimicrobial agent, which coincides with the presented results. Its antifungal and antibacterial potential had been tested in laboratory conditions as well as in the clinical trials [54]. 

According to the obtained results on the efficacy of the acquired materials containing ozonated olive oil (nHAp mixed with ozonated olive oil, mixed with olive oil and doped with Cu^2+^ ions, pure ozonated olive oil, respectively), their addition to microbiological samples causes the highest percentage reduction when compared to materials without ozonated olive oil (I and II). When analyzing the given data, there is an obvious conclusion that the highest efficacy of olive is presented in samples of *S. mutans*, especially in nHAp of a concentration of 0.1% for nHAp with the addition of ozonated olive oil, and also in 1% nHAp concentration for nHAp doped with Cu^2+^ ions and ozonated olive oil. The total growth reduction of *S. mutans* strains is visible in nHAp doped with Cu^2+^ ions and ozonated olive oil 4 h after application, as nearly 100% efficacy can be observed; however, in nHAp with ozonated olive oil, it occurs after 24 h. A complete reduction of *S. mutans* cells was observed after incubating this bacterial strain in the presence of ozonated olive oil. This study allows deducing that the *S. mutans* percentage reduction can reach the highest value among the measured microorganisms when it is mixed with nHAp doped with Cu^2+^ ions and loaded with ozonated olive oil. In the case of ozonated olive oil, the lowest percentage reduction value of *S. mutans* is present 24 h after the use of 0.1% nHAp with the addition of ozonated olive. Additionally, olive efficacy was also measured by the number of colonies concentration (thousand CFU/mL) concerning *S. mutans*. The value was the same—0.018 ± 0.008 when the species were mixed with selected materials at a weight of 1% (nHAp with ozonated olive oil, nHAp with Cu^2+^ and ozonated olive oil, ozonated olive oil). Interestingly, when the CFU/mL of *S. mutans* was compared after contact with selected materials at a weight of 0.1%, each material concerning olive lacked growth except for nHAp doped with Cu^2+^ ions and ozonated olive oil, which had 2 × 10^6^ ± 0.01.

The lowest percentage reduction value of ozonated olive oil inflicts *L. rhamnosus* when it is mixed with 0.1% nHAp with the addition of ozonated olive oil. Even when mixed with pure ozonated olive oil, *L. rhamnosus* represents the lowest percentage reduction among every tested microorganism strain. The CFU/mL of *L. rhamnosus* had the same value as *S. mutans* in most cases (0.018 ± 0.008), but this was only after contact with pure ozonated olive oil at 0.1% sample weight. On the other hand, for nHAp with ozonated olive oil as well as nHAp doped with Cu^2+^ ions and ozonated olive oil, the values were higher, ranging from 6.07 × 10^8^ to 1.12 × 10^9^.

The colony-forming unit of *L. rhamnosus* is less optimistic after contact with ozonated olive oil materials (nHAp with ozonated olive oil, nHAp with Cu^2+^ with ozonated olive oil, and ozonated olive oil alone). At a sample weight of 1%, values ranged between 1 × 10³ and 1 × 10⁶.

By analyzing data acquired by the authors in the study, it can be concluded that the percentage reduction of the measured microorganisms and CFU/mL. have the most positive results for materials containing ozonated olive oil (nHAp with the addition of ozonated olive oil, nHAp doped with Cu^2+^ ions and loaded with ozonated olive oil, ozonated olive oil). The percentage reduction in value after contact with pure ozonated olive oil is constantly high, ranging between 71 and 99%, and the highest results are obtained after 24 h of cultivation, ranging between 94 and 99% (see Tables S9 and S10). Similar results concerning a high efficacy of ozonated olive oil against these microorganisms were obtained by several other authors [54,55]. The CFU/mL of olive itself, with materials at a weight of 0.1%, has the best efficacy for each measured microorganism. On the other hand, at a sample weight of 1% nHAp, the same result is acquired only in the case of *S. mutans*, while the worst result is obtained for *L. rhamnosus*—1 × 10^6^ ± 10.14.

Nanohydroxyapatite doped with Cu^2+^ ions or ozonated olive oil may limit the oral microbial activity. Moreover, it is successfully applied in dentine hypersensitivity treatment and maxillofacial bones regeneration. Doping it with Cu^2+^ ions or ozonated olive oil may enhance its antimicrobial characteristics and limit the postoperative complications.

## 5. Conclusions

Calcium hydroxyapatite and calcium hydroxyapatite doped with Cu^2+^ ions have been successfully synthetized by using the wet chemistry method. The obtained nanocrystals have been functionalized with ozonated olive oil, which resulted in the formation of a novel medical composition. In vitro screening of microorganism strains according to their activity in various experimental conditions may be a valuable method that could precede clinical efficacy treatments. The highest efficacy was observed for ozonated olive oil and nanocrystalline Cu^2+^-doped nHAp followed by undoped nHAp. The obtained results indicate that 10 times higher concentrations of pure as well as doped nHAp have better antimicrobial activity. *Streptococcus mutans* had the highest sensitivity, while *Lactobacillus rhamnosus* had the lowest. Pure ozonated olive oil had the highest antimicrobial efficacy. 

In the next stage of the study, the authors plan to evaluate the activity of the nHAp against the biofilms in order to complete a whole view of the bacteria cell properties as well as the effectiveness of the antimicrobial compounds.

## Figures and Tables

**Table 1:** Values of Minimal Inhibitory Concentration (MIC) of nHAp for analyzed strains.

Species	MIC (µg/ mL)			
	I	II	III	IV
*Candida albicans*	>5000	5000	5000	5000
*Streptococcus mutans*	>5000	>5000	5000	5000
*Lactobacillus rhamnosus*	>5000	>5000	>5000	>5000

I—nHAp; II—Cu^2+^-doped nHAp; III—nHAp with the addition of ozonated olive oil; IV—nHAp doped with Cu^2+^ and loaded with ozonated olive oil.

## Supplementary Materials

The following are available online at https://www.mdpi.com/2079-4991/10/10/1997/s1. Table S1: The effect of pure and doped nHAp on the analysed strains (optical density (OD) 595 nm); Table S2: Inhibition of the growth of microbial cells, %; Table S3: Average CFU/mL and standard deviation (M ± SD) for the tested strains with selected materials at a concentration of 0.1% nHAp; Table S4: Average CFU/mL and standard deviation (M ± SD) for the tested strains with selected materials with concentration of 1% nHAp; Table S5: Evaluation of the antimicrobial properties of ozonated olive oil (Inhibition % equals 99.99% for each strain); Table S6: Results of comparisons of *Candida albicans* colonies after contact with tested materials (C—growth control, I—nHAp, II—Cu^2+^-doped nHAp, III—nHAp with the addition of ozonated olive oil, IV—nHAp doped with Cu^2+^ ions and loaded with ozonated olive oil, V—ozonated olive oil) for weight samples of 0.1 and 1% (M—average value); Table S7: Results of comparisons of *Lactobacillus rhamnosus* colonies after contact with tested materials (C—growth control, I—nHAp, II—Cu^2+^-doped nHAp, III—nHAp with the addition of ozonated olive oil, IV—nHAp doped with Cu^2+^ ions and loaded with ozonated olive oil, V—ozonated olive oil) for weight samples of 0.1 and 1% (M—average value); Table S8: Results of comparisons of *Streptococcus mutans* colonies after contact with tested materials (C—growth control, I—nHAp, II—Cu^2+^-doped nHAp, III—nHAp with the addition of ozonated olive oil, IV—nHAp doped with Cu^2+^ ions and loaded with ozonated olive oil, V—ozonated olive oil) for weight samples of 0.1 and 1% (M—average value); Table S9: Influence of 0.1% hydroxyapatite on microbial strains: *C. albicans*, *L. rhamnosus* i *S. mutans* (Inhibition growth%); Table S10: Influence of 1% hydroxyapatite on microbial strains: *C. albicans*, *L. rhamnosus* i *S. mutans* (Inhibition growth%).
