# Supplementary material for: The Influence of Ozonated Olive Oil-Loaded and Copper-Doped Nanohydroxyapatites on Planktonic Forms of Microorganisms
Source: Nanomaterials (Basel). 2020 Oct 10;10(10):1997. doi: 10.3390/nano10101997 (PMC7650683; doi:10.3390/nano10101997)
Supplement: Supplementary file 1 [file nanomaterials-10-01997-s001.docx]

Supplementary Materials

The Influence of Ozonated Olive Oil-Loaded and Copper-Doped Nanohydroxyapatites on Planktonic Forms of Microorganisms

Wojciech Zakrzewski ^1^, Maciej Dobrzynski ^2^, Joanna Nowicka ^3^, Magdalena Pajaczkowska ^3^, Maria Szymonowicz ^1^, Sara Targonska ^4^, Paulina Sobierajska ^4^, Katarzyna Wiglusz ^5^, Wojciech Dobrzynski ^6^, Adam Lubojanski ^1^, Sebastian Fedorowicz ^3^, Zbigniew Rybak ^1^ and Rafal J. Wiglusz ^4,^*

^1^ Department of Experimental Surgery and Biomaterial Research, Wroclaw Medical University, Bujwida 44, 50-345 Wroclaw, Poland; wojciech.zakrzewski@student.umed.wroc.pl (W.Z.); maria.szymonowicz@umed.wroc.pl (M.S.); adam.lubojanski@student.umed.wroc.pl (A.L.); zbigniew.rybak@umed.wroc.pl (Z.R.)

^2^ Department of Conservative Dentistry and Pedodontics, Wroclaw Medical University, Krakowska 26, 50-425 Wroclaw, Poland; maciej.dobrzynski@umed.wroc.pl

^3^ Department of Microbiology, Wroclaw Medical University, Chalubinskiego 4, 50-368 Wroclaw, Poland; joanna.nowicka@umed.wroc.pl (J.N.); magdalena.pajaczkowska@umed.wroc.pl (M.P.); sebastian.fedorowicz@student.umed.wroc.pl (S.F.)

^4^ Institute of Low Temperature and Structure Research, Polish Academy of Sciences, Okolna 2, 50-422 Wroclaw, Poland; s.targonska@intibs.pl (S.T.); p.sobierajska@intibs.pl (P.S.)

^5^ Department of Analytical Chemistry, Wroclaw Medical University, Borowska 211 A, 50-566 Wroclaw, Poland; katarzyna.wiglusz@umed.wroc.pl

^6^ Student Scientific Circle at the Department of Dental Materials, School of Medicine with the Division of Dentistry in Zabrze, Medical University of Silesia in Katowice, Akademicki Sq. 17, 41-902 Bytom, Poland; wojt.dobrzynski@wp.pl

* Correspondence: r.wiglusz@intibs.pl; Tel.: +48-71-3954-159; Fax: +48-71-344-10-29

**Table S1.** The effect of pure and doped nHAp on the analysed strains (optical density (OD) 595 nm).

| **Species** | **Time (h)** | **0.1%** | | | | | **1%** | | | | | **V** |
| --- | --- | --- | --- | --- | --- | --- | --- | --- | --- | --- | --- | --- |
|  |  | **C** | **I** | **II** | **III** | **IV** | **C** | **I** | **II** | **III** | **IV** |  |
| C. albicans | 4 | 0.435 | 0.430 | 0.235 | 0.139 | 0.058 | 0.391 | 0.354 | 0.22 | 0.104 | 0.064 | 0.061 |
| S. mutans |  | 0.182 | 0.180 | 0.097 | 0.069 | 0.005 | 0.203 | 0.193 | 0.047 | 0.040 | 0.001 | 0.059 |
| L. rhamnosus |  | 0.207 | 0.180 | 0.128 | 0.087 | 0.1 | 0.212 | 0.197 | 0.161 | 0.121 | 0.097 | 0.004 |
| C. albicans | 24 | 1.143 | 0.982 | 0.899 | 0.212 | 0.126 | 1.068 | 0.671 | 0.539 | 0.414 | 0.156 | 0.069 |
| S. mutans |  | 0.448 | 0.417 | 0.263 | 0.287 | 0.114 | 0.517 | 0.480 | 0.417 | 0.02 | 0.096 | 0.022 |
| L. rhamnosus |  | 1.369 | 1.242 | 1.212 | 0.978 | 0.514 | 1.380 | 1.230 | 1.309 | 0.362 | 0.163 | 0.003 |

C—growth control; I—nHAp; II—Cu^2+^-doped nHAp; III—nHAp with the addition of ozonated olive; IV—nHAp doped with Cu^2+^ and loaded with ozonated olive; V—ozonated olive; SD ± 0.05.

**Table S2*.*** Inhibition of the growth of microbial cells, %*.*

| **Species** | **Time**  **(h)** | **0.1%** | | | | **1%** | | | | **V** |
| --- | --- | --- | --- | --- | --- | --- | --- | --- | --- | --- |
|  |  | **I** | **II** | **III** | **IV** | **I** | **II** | **III** | **IV** |  |
| C. albicans | 4 | 1 | 46 | 68 | 87 | 9 | 44 | 73 | 84 | 84 |
| S. mutans |  | 1 | 47 | 62 | 97 | 5 | 77 | 80 | 100 | 71 |
| L. rhamnosus |  | 13 | 38 | 58 | 52 | 7 | 24 | 43 | 54 | 98 |
| C. albicans | 24 | 14 | 21 | 81 | 89 | 37 | 50 | 61 | 52 | 94 |
| S. mutans |  | 7 | 41 | 36 | 75 | 7 | 19 | 96 | 81 | 96 |
| L. rhamnosus |  | 9 | 11 | 29 | 62 | 11 | 5 | 74 | 88 | 99 |

I—nHAp; II—Cu^2+^-doped nHAp; III—nHAp with the addition of ozonated olive; IV—nHAp doped with Cu^2+^ and loaded with ozonated olive; V—ozonated olive; SD ± 0.1.

**Table S3.** Average CFU/mL and standard deviation (M ± SD) for the tested strains with selected materials at a concentration of 0.1% nHAp.

| **Materials** | **Microorganisms species** | | |
| --- | --- | --- | --- |
|  | ***Candida***  ***albicans***  **CFU/mL × 10^3^**  **M ± SD** | ***Lactobacillus***  ***rhamnosus***  **CFU/mL × 10^3^**  **M ± SD** | ***Streptococcus***  ***mutans***  **CFU/mL × 10^3^**  **M ± SD** |
| C*—*growth control | 146,000 ± 8,485 | 501,000 ± 55,154 | 3,500 ± 707 |
| I—nHAp | 320,500 ± 707 | 1,123,500 ± 4950 | 81,500 ± 71 |
| II*—*Cu^2+^-doped nHAp | 229,500 ± 707 | 1,675,000 ± 7071 | 0.018 ± 0.008 |
| III—nHAp with the addition of ozonated olive oil | 140 ± 0.01 | 607,000 ± 0.01 | 0.018 ± 0.008 |
| IV—nHAp doped with Cu^2+^ and loaded with ozonated olive oil | 5000 ± 0.01 | 1,120,500 ± 707 | 2000 ± 0.01 |

M*—*average, SD*—*standard deviation.

**Table S4.** Average CFU/mL and standard deviation (M ± SD) for the tested strains with selected materials with concentration of 1% nHAp.

| **Materials** | **Microorganisms species** | | |
| --- | --- | --- | --- |
|  | ***Candida***  ***albicans***  **CFU/mL × 10^3^**  **M ± SD** | ***Lactobacillus***  ***rhamnosus***  **CFU/mL × 10^3^**  **M ± SD** | ***Streptococcus***  ***mutant***  **CFU/mL × 10^3^**  **M ± SD** |
| C*—*growth control | 90,000 ± 1414 | 1,490,000 ± 410,122 | 24,900 ± 3394 |
| I—nHAp | 179,000 ± 103,238 | 3,360,000 ± 593,970 | 61,300 ± 7495 |
| II—Cu^2+^-doped nHAp | 99,000 ± 55,154 | 1,330,000 ± 353,553 | 0.018 ± 0.008 |
| III—nHAp with the addition of ozonated olive oil | 140.0 ± 0.1 | 113.0 ± 42 | 0.018 ± 0.008 |
| IV—nHAp doped with Cu^2+^ and loaded with ozonated olive oil | 2600 ± 426 | 1.0 ± 0.1 | 0.018 ± 0.008 |

**Table S5.** Evaluation of the antimicrobial properties of ozonated olive oil (Inhibition % equals 99.99% for each strain).

| **Species** | **Growth control**  **CFU/mL × 10^3^**  **M ± SD** | **Ozonated olive oil**  **CFU/mL × 10^3^**  **M ± SD** |
| --- | --- | --- |
| *Candida albicans* | 90,000 ± 1414 | 4.0 ± 1.4 |
| *Streptococcus mutans* | 24,900 ± 3394 | 0.018 ± 0.008 |
| *Lactobacillus rhamnosus* | 1,490,000 ± 410,122 | 1000 ± 10.14 |

**Table S6.** Results of comparisons of *Candida albicans* colonies after contact with tested materials (C—growth control, I—nHAp, II—Cu^2+^-doped nHAp, III—nHAp with the addition of ozonated olive oil, IV—nHAp doped with Cu^2+^ ions and loaded with ozonated olive oil, V*—*ozonated olive oil**)** for weight samples of 0.1 and 1% (M—average value).

|  | ***Candida albicans* colonies. weight samples of 0.1%** | | | | | |
| --- | --- | --- | --- | --- | --- | --- |
|  | **C**  **M = 146,000** | **I**  **M = 320,500** | **II**  **M = 229,500** | **III**  **M = 140** | **IV**  **M = 5000** | **V**  **M = 0.01** |
| **C** | × | *p* < 0.001 | *p* < 0.001 | *p* < 0.001 | *p* < 0.001 | *p* < 0.001 |
| **I** | *p* < 0.001 | × | *p* < 0.001 | *p* < 0.001 | *p* < 0.001 | *p* < 0.001 |
| **II** | *p* < 0.001 | *p* < 0.001 | × | *p* < 0.001 | *p* < 0.001 | *p* < 0.001 |
| **III** | *p* < 0.001 | *p* < 0.001 | *p* < 0.001 | × | *p* = 0.726 | *p* = 1.000 |
| **IV** | *p* < 0.001 | *p* < 0.001 | *p* < 0.001 | *p* > 0.05 | × | *p* = 0.711 |
| **V** | *p* < 0.001 | *p* < 0.001 | *p* < 0.001 | *p* > 0.05 | *p* > 0.05 | × |
|  | ***Candida albicans* colonies. weight samples of 1.0%** | | | | | |
|  | **C**  **M = 90,000** | **I**  **M = 179,000** | **II**  **M = 99,000** | **III**  **M = 140** | **IV**  **M = 2600** | **V**  **M = 4.0** |
| **C** | × | *p* = 0.112 | *p* = 0.857 | *p* = 0.109 | *p* = 0.117 | *p* = 0.109 |
| **I** | *p* > 0.05 | × | *p* = 0.145 | *p* = 0.010 | *p* = 0.010 | *p* = 0.010 |
| **II** | *p* > 0.05 | *p* > 0.05 | × | *p* = 0.084 | *p* = 0.090 | *p* = 0.084 |
| **III** | *p* > 0.05 | *p* < 0.05 | *p* > 0.05 | × | *p* = 0.961 | *p* = 0.998 |
| **IV** | *p* > 0.05 | *p* < 0.05 | *p* > 0.05 | *p* > 0.05 | × | *p* = 0.958 |
| **V** | *p* > 0.05 | *p* < 0.05 | *p* > 0.05 | *p* > 0.05 | *p* > 0.05 | × |

**Table S7.** Results of comparisons of *Lactobacillus rhamnosus* colonies after contact with tested materials (C—growth control, I—nHAp, II—Cu^2+^-doped nHAp, III—nHAp with the addition of ozonated olive oil, IV—nHAp doped with Cu^2+^ ions and loaded with ozonated olive oil, V—ozonated olive oil**)** for weight samples of 0.1 and 1% (M—average value).

|  | ***Lactobacillus rhamnosus* colonies. weight samples 0.1%** | | | | | |
| --- | --- | --- | --- | --- | --- | --- |
|  | **C**  **M = 501,000** | **I**  **M = 1,123,500** | **II**  **M = 1,675,000** | **III**  **M = 607,000** | **IV**  **M = 1,120,500** | **V**  **M = 0.02** |
| **C** | × | *p* < 0.001 | *p* < 0.001 | *p* < 0.001 | *p* < 0.001 | *p* < 0.001 |
| **I** | *p* < 0.001 | × | *p* < 0.001 | *p* < 0.001 | *p* = 0.900 | *p* < 0.001 |
| **II** | *p* < 0.001 | *p* < 0.001 | × | *p* < 0.001 | *p* < 0.001 | *p* < 0.001 |
| **III** | *p* < 0.001 | *p* < 0.001 | *p* < 0.001 | × | *p* < 0.001 | *p* < 0.001 |
| **IV** | *p* < 0.001 | *p* > 0.05 | *p* < 0.001 | *p* < 0.001 | × | *p* < 0.001 |
| **V** | *p* < 0.001 | *p* < 0.001 | *p* < 0.001 | *p* < 0.001 | *p* < 0.001 | × |
|  | ***Lactobacillus rhamnosus* colonies. weight samples 1.0%** | | | | | |
|  | **C**  **M = 1,490,000** | **I**  **M = 3,360,000** | **II**  **M = 1,330,000** | **III**  **M = 113** | **IV**  **M = 1** | **V**  **M = 1000** |
| **C** | × | *p* = 0.001 | *p* = 0.643 | *p* = 0.004 | *p* = 0.004 | *p* = 0.004 |
| **I** | *p* < 0.01 | × | *p* = 0.001 | *p* < 0.001 | *p* < 0.001 | *p* = 0.007 |
| **II** | *p* > 0.05 | *p* < 0.01 | × | *p* = 0.007 | *p* = 0.007 | *p* = 0.007 |
| **III** | *p* < 0.01 | *p* < 0.001 | *p* < 0.01 | × | *p* = 1.000 | *p* = 0.998 |
| **IV** | *p* < 0.01 | *p* < 0.001 | *p* < 0.01 | *p* > 0.05 | × | *p* = 0.998 |
| **V** | *p* < 0.01 | *p* < 0.01 | *p* < 0.01 | *p* > 0.05 | *p* > 0.05 | × |

**Table S8.** Results of comparisons of *Streptococcus mutans* colonies after contact with tested materials (C—growth control, I—nHAp, II—Cu^2+^-doped nHAp, III—nHAp with the addition of ozonated olive oil, IV—nHAp doped with Cu^2+^ ions and loaded with ozonated olive oil, V—ozonated olive oil**)** for weight samples of 0.1 and 1% (M—average value).

|  | ***Streptococcus mutans* colonies. weight samples 0.1%** | | | | | |
| --- | --- | --- | --- | --- | --- | --- |
|  | **C**  **M = 3500** | **I**  **M = 81,500** | **II**  **M = 0.02** | **III**  **M = 0.02** | **IV**  **M = 2000** | **V**  **M = 0.02** |
| **C** | × | *p* < 0.001 | *p* < 0.001 | *p* < 0.001 | *p* = 0.002 | *p* < 0.001 |
| **I** | *p* < 0.001 | × | *p* < 0.001 | *p* < 0.001 | *p* < 0.001 | *p* < 0.001 |
| **II** | *p* < 0.001 | *p* < 0.001 | × | *p* = 1.000 | *p* < 0.001 | *p* = 1.000 |
| **III** | *p* < 0.001 | *p* < 0.001 | *p* > 0.05 | × | *p* < 0.001 | *p* = 1.000 |
| **IV** | *p* < 0.001 | *p* < 0.001 | *p* < 0.001 | *p* < 0.001 | × | *p* < 0.001 |
| **V** | *p* < 0.001 | *p* < 0.001 | *p* > 0.05 | *p* > 0.05 | *p* < 0.001 | × |
|  | ***Streptococcus mutans* colonies. weight samples 1.0%** | | | | | |
|  | **C**  **M = 24,900** | **I**  **M = 61,300** | **II**  **M = 0.02** | **III**  **M = 0.02** | **IV**  **M = 0.02** | **V**  **M = 0.02** |
| **K** | × | *p* < 0.001 | *p* < 0.001 | *p* < 0.001 | *p* = 0.002 | *p* < 0.001 |
| **I** | *p* < 0.001 | × | *p* < 0.001 | *p* < 0.001 | *p* < 0.001 | *p* < 0.001 |
| **II** | *p* < 0.001 | *p* < 0.001 | × | *p* = 1.000 | *p* = 1.000 | *p* = 1.000 |
| **III** | *p* < 0.001 | *p* < 0.001 | *p* > 0.05 | × | *p* = 1.000 | *p* = 1.000 |
| **IV** | *p* < 0.001 | *p* < 0.001 | *p* > 0.05 | *p* > 0.05 | × | *p* = 1.000 |
| **V** | *p* < 0.001 | *p* < 0.001 | *p* > 0.05 | *p* > 0.05 | *p* > 0.05 | × |

**Table S9.** Influence of 0.1% hydroxyapatite on microbial strains: *C. albicans*, *L. rhamnosus* i *S. mutans* (Inhibition growth%).

| **Materials** | **Microorganisms species**  **(% inhibition growth)** | | |
| --- | --- | --- | --- |
|  | ***Candida***  ***albicans*** | ***Lactobacillus***  ***rhamnosus*** | ***Streptococcus***  ***mutant*** |
| I—nHAp | No reduction | No reduction | No reduction |
| II—Cu^2+^-doped nHAp | No reduction | No reduction | 100 |
| III—nHAp with the addition of ozonated olive oil | 99.4 | 21 | 100 |
| IV—nHAp doped with Cu^2+^ and loaded with ozonated olive oil | 96.58 | No reduction | 43 |

I—nHAp; II—Cu^2+^-doped nHAp; III—nHAp with the addition of ozonated olive oil; IV—nHAp doped with Cu^2+^ ions and loaded with ozonated olive oil.

**Table S10.** Influence of 1% hydroxyapatite on microbial strains: *C. albicans*, *L. rhamnosus* i *S. mutans* (Inhibition growth%).

| **Materials** | **Microorganisms species**  **(% inhibition growth)** | | |
| --- | --- | --- | --- |
|  | ***Candida***  ***albicans*** | ***Lactobacillus***  ***rhamnosus*** | ***Streptococcus***  ***mutant*** |
| I—nHAp | No reduction | No reduction | No reduction |
| II—Cu^2+^-doped nHAp | No reduction | 11 | 100 |
| III—nHAp with the addition of ozonated olive oil | 99.9 | 99.99 | 100 |
| IV—nHAp doped with Cu^2+^ and loaded with ozonated olive oil | 97.2 | 99.3 | 100 |

I—nHAp; II—Cu^2+^-doped nHAp; III—nHAp with the addition of ozonated olive oil; IV—nHAp doped with Cu^2+^ ions and loaded with ozonated olive oil.
